# Oncological Outcomes of Epirubicin-based Hyperthermic Intravesical Chemotherapy in Bacillus Calmette-Guérin (BCG)-naïve Patients and Patients with BCG Failure: A Multicenter Retrospective Observational Study

**DOI:** 10.1016/j.euros.2026.02.008

**Published:** 2026-02-23

**Authors:** Nicolas Arnold, Lukas Koneval, Julien Blanc, Ilaria Lucca, Mihai Dorin Vartolomei, Laila Schneidewind, Nicola Giudici, George N. Thalmann, Bernhard Kiss, Beat Roth

**Affiliations:** a Department of Urology, University Hospital Bern, Bern, Switzerland; b Department of Urology, University Hospital Lausanne, Lausanne, Switzerland; c Institute for University Doctoral Studies, Iuliu Hatieganu University of Medicine and Pharmacy, Cluj-Napoca, Romania

**Keywords:** Bladder cancer, Hyperthermic intravesical chemotherapy, Epirubicin, Recurrence, Progression, Cystectomy

## Abstract

**Background and objective:**

Intravesical instillation of bacillus Calmette-Guérin (BCG) is the standard therapy for patients with intermediate-risk or high-risk non–muscle-invasive bladder cancer. In cases of BCG failure, device-assisted hyperthermic intravesical chemotherapy (HIVEC) using epirubicin can be offered to patients who are unfit for or decline radical cystectomy. The aim of this study was to investigate long-term oncological outcomes for patients treated with HIVEC in a first-line or second-line setting.

**Methods:**

We retrospectively analyzed our prospective database of patients who underwent HIVEC with epirubicin between November 2016 and December 2024 at two Swiss university centers. Survival was assessed using Kaplan-Meier curves and log-rank tests. Cox regression models were used to explore associations between clinicopathological variables and time-to-event outcomes.

**Key findings and limitations:**

Of the 88 patients included, 23 (26.1%) received HIVEC as first-line treatment and 65 (73.9%) after BCG failure. Median follow-up was 38 mo (interquartile range 22–54). Intravesical recurrence was observed in 26 patients (29.6%), with 1-yr and 2-yr intravesical recurrence–free survival rates of 84.1% and 77.3%, respectively. Extravesical recurrence in the upper urinary tract or prostatic urethra occurred in 21 patients (23.9%). Progression to muscle-invasive or metastatic disease due to intravesical recurrence was observed in eight patients (9.1%), with 1-yr and 2-yr progression-free survival rates of 96.6% and 93.2%, respectively.

**Conclusions and clinical implications:**

Device-assisted epirubicin-based HIVEC yielded encouraging long-term oncological outcomes both for first-line therapy and after BCG failure. Extravesical recurrences were observed at a relatively high rate in the group with BCG failure, which highlights the importance of monitoring for extravesical disease in future studies and follow-up planning. Prospective studies are needed to confirm the efficacy of epirubicin-based HIVEC, particularly in first-line settings.

**Patient summary:**

We tested in-bladder treatment with a heated solution of a chemotherapy drug for patients with recurrence of non–muscle-invasive bladder cancer after failure of BCG (bacillus Calmette-Guérin) therapy. Our results show promising cancer control rates after BCG failure, and suggest that this treatment may also be a valuable option when BCG is unavailable and in patients for whom BCG is not suitable.

## Take Home Message

Hyperthermic intravesical chemotherapy using epirubicin showed encouraging oncological results when given after bacillus Calmette-Guérin (BCG) failure and appears to be a potential treatment alternative in a first-line setting when BCG is not available or contraindicated.

## 1 Introduction

Bladder cancer is one of the ten most commonly diagnosed cancers worldwide [1]. In 70% of cases, it is diagnosed as non–muscle-invasive bladder cancer (NMIBC), which generally suggests favorable oncological outcomes [2]. However, NMIBC represents a highly heterogeneous group of tumors, ranging from low-grade Ta with low aggressiveness, to highly aggressive high-grade T1 disease. This heterogeneity is reflected in considerable risk of recurrence and progression after initial transurethral resection of bladder tumor. Recurrence rates range from 15% to 31% at 1 yr, and from 61% to 78% at 5 yr, while progression rates range from 0.2% to 0.8% at 1 y and from 17% to 45% at 5 yr [3].

The European Organization for Research and Treatment of Cancer (EORTC) and the European Association of Urology (EAU) have both developed scoring models that stratify patients with NMIBC into prognostic risk groups for recurrence and progression. To reduce the risk of recurrence and of progression to muscle-invasive disease, adjuvant intravesical therapy should be considered, depending on the tumor characteristics [4]. For patients with intermediate-risk or high-risk NMIBC, intravesical immunotherapy with bacillus Calmette-Guérin (BCG) remains the most effective conservative treatment option following complete resection [5], [6], [7], [8]. Nevertheless, up to 30–40% of patients experience disease recurrence after an initial response (BCG-relapsing disease) [9], while others have BCG-unresponsive NMIBC that shows either disease persistence (BCG-refractory) or the development of high-grade tumor within 6 mo or carcinoma in situ (CIS) within 12 mo of adequate BCG treatment [4]. These patients are unlikely to benefit from further BCG therapy and should be offered radical cystectomy [4].

However, some patients are unfit for cystectomy, while others are unwilling to undergo major surgery. Furthermore, since 2012, regular BCG shortages have created an urgent need for alternative treatment strategies [10]. Some patients exhibit intolerance or relative contraindications, such as immunosuppression, that preclude BCG therapy [11]. Therefore, several centers have evaluated alternative intravesical therapies [12], [13], [14], [15], [16], [17], [18], [19], [20]. Among these, combinations of cytotoxic agents, most commonly mitomycin C (MMC), with intravesical hyperthermia (device-assisted hyperthermic intravesical chemotherapy; HIVEC) have been explored since the late 1990s [21]. Hyperthermia at 40–44°C enhances the cytotoxic effect of chemotherapeutic agents [22], [23]. Although HIVEC using MMC and a microwave-induced system to heat the bladder wall has shown promising oncological results [24], [25], treatment has been associated with frequent adverse events, with discontinuation rates of up to 38% [26]. In addition, several studies are currently investigating novel agents for BCG-resistant disease, some of which have already been approved by the US Food and Drug Administration [27], [28], [29].

To overcome these drawbacks, we evaluated an alternative delivery system based on conductive heating (UniThermia) with a different chemotherapeutic agent, epirubicin, an anthracycline that inhibits DNA replication, transcription, and repair, in an optimized treatment setting [30].

## 2 Patients and methods

### 2.1 Study design

We conducted a retrospective observational analysis of a prospectively maintained database for two Swiss University Hospitals (Bern and Lausanne) between November 2016 and December 2024. Institutional board approval was obtained from the ethics committee of the Canton of Bern, Switzerland (protocol number 121/08 [31]). The study is reported according to the Strengthening the Reporting of Observational Studies in Epidemiology (STROBE) guidelines and was conducted in accordance with ethical standards laid down in the 1964 Declaration of Helsinki and its later amendments. All patients provided informed consent before study inclusion. No sensitivity analyses were performed owing to sample size constraints.

### 2.2 Patients

During the first major BCG shortage in Switzerland in 2016, we established a prospective protocol for the administration of HIVEC using epirubicin and conductive heating. Patients underwent transurethral resection or bladder mapping biopsies in cases with positive cytology but no macroscopic tumor on white-light and/or blue-light cystoscopy. Patients with pT1 tumors or high-grade pTa tumors underwent a second transurethral resection 2–4 wk after initial resection to exclude muscle-invasive or residual disease other than carcinoma in situ (CIS). Patients with intermediate-risk, high-risk, or very high-risk urothelial NMIBC according to the EAU classification were eligible for inclusion. Patients qualified if (1) radical cystectomy was indicated because of high-grade recurrence or persistence of high-grade disease/CIS after prior intravesical BCG therapy, but were either ineligible for cystectomy because of comorbidities or refused major surgery; (2) HIVEC was initiated in BCG-naïve patients as first-line treatment owing to BCG shortage; (3) BCG was contraindicated (eg, immunosuppression); or (4) HIVEC was applied in the setting of NMIBC low-grade recurrence. Exclusion criteria comprised muscle-invasive disease, allergy or intolerance to epirubicin, and refusal of the HIVEC protocol. To reduce cohort heterogeneity, patients with nonurothelial histology were also excluded. The study size was determined by the number of consecutive eligible patients treated during the study period. Missing data were minimal and handled using a complete-case analysis.

### 2.3 Treatment

Device-assisted HIVEC was performed using a UniThermia heating system (Elmedical, Hod-Hasharon, Israel) with epirubicin (50 mg diluted in 50 ml of 0.9% saline) heated to 43°C for 50 min. Patients were treated with 4 g of sodium bicarbonate during the 12 h preceding HIVEC to alkalinize their urine (pH target ≥6) and thus minimize biodegradation. The sodium bicarbonate dosage was individually adjusted after each treatment according to urinary pH values measured. Patients were instructed to not drink more than 2 dl of fluids within 4 h preceding HIVEC to keep drug dilution as low as possible.

### 2.4 Schedule

Treatment consisted of an induction cycle of six weekly instillations, followed by a maintenance cycle with six monthly instillations. Follow-up included cystoscopy and cytology every 3 mo for 2 yr, and every 6 mo thereafter for at least 5 yr in recurrence-free patients. Suspected recurrences were confirmed via transurethral resection. In cases with positive cytology without visible lesions, random bladder and prostatic urethra biopsies, selective upper tract cytology, and diagnostic ureteroscopy were performed as indicated. Abdominal computed tomography scans were scheduled at 6, 12, 24, and 60 mo, or if recurrence was suspected.

### 2.5 Outcome measurements

The study objective was to characterize oncological outcomes in patients treated with epirubicin-based HIVEC. We aimed to estimate time-to-event outcomes to explore associations between clinicopathological variables and the risk of recurrence, progression, and death. The primary endpoint was intravesical recurrence-free survival (RFS), defined as histologically confirmed NMIBC or CIS. Secondary endpoints were progression-free survival (PFS), defined as muscle-invasive and/or metastatic disease originating from bladder cancer, and extravesical RFS (upper urinary tract or prostatic urethra). Further exploratory endpoints were disease-free survival (DFS), cancer-specific survival (CSS), overall survival (OS), and radical cystectomy. Follow-up commenced on the date of first HIVEC administration. Patients without recurrence were censored for RFS at the last cystoscopy performed. For PFS, extravesical RFS, DFS, CSS, and OS, censoring occurred at the time of the last follow-up visit at our outpatient clinic without evidence of an event. A cause-specific approach was used, and competing events were treated as censoring events. Administrative censoring took place on December 31, 2024.

### 2.6 Statistical analysis

Results are summarized as the absolute frequency and percentage for categorical variables, and as the median with interquartile range (IQR) for continuous variables. Time-to-event outcomes were analyzed using the Kaplan-Meier method and log-rank tests to characterize oncological outcomes over time. The study was designed as a purely descriptive and prognostic analysis. Accordingly, univariate and multivariable Cox regression analyses were used to explore associations rather than casual effects of clinicopathological variables, including: age (continuous); sex (male vs female); highest stage (pTa vs pT1 vs CIS only) and grade (low-grade vs high-grade) before and at the time of HIVEC; CIS (yes vs no) before and at the time of HIVEC; BCG failure (yes vs no); type of BCG failure (relapsing vs refractory); BCG unresponsiveness (yes vs no); EORTC recurrence and progression scores (continuous) and EAU risk category (intermediate-risk, high-risk, very high-risk) at the time of HIVEC. Covariates for multivariable Cox models were chosen pragmatically: age and sex were included a priori, and additional variables with *p* ≤ 0.05 on univariate Cox analyses were entered into the multivariable model. Age and EORTC scores were modeled continuously; other covariates were coded categorically as specified. Given the multiple endpoints, the primary endpoint was prioritized for inference. Analyses of secondary and exploratory endpoints were considered hypothesis-generating; no formal adjustment for multiplicity was performed, and *p* values for these endpoints should be interpreted descriptively. Results were reported as the hazard ratio (HR) with 95% confidence interval (CI). All statistical analyses were performed using Stata version 11 (StataCorp, College Station, TX, USA), with *p* < 0.05 considered statistically significant.

## 3 Results

### 3.1 General results

A total of 88 patients were included, of whom 75 (85.2%) were male and 13 (14.8%) were female. The median age was 71 yr (IQR 66–77) and median follow-up was 38 mo (IQR 22–54). Six patients were lost to follow-up. Fifteen patients (17.0%) had primary tumors, while 73 (83.0%) had recurrent disease with a median of two recurrences (IQR 1–3). HIVEC was administered as first-line therapy in 23 BCG-naïve patients (26.1%) and after BCG failure in 65 patients (73.9%). Among the latter, 43/65 (66.1%) had completed one full induction cycle of BCG. In addition, of 65 patients, 16 (24.6%) had received one, and six (9.2%) had received two or more maintenance cycles of BCG.

Of 65 BCG failures, 23 (35.4%) were refractory and 42 (64.6%) relapsing, with 31 cases (47.7%) classified as BCG-unresponsive. The median time from the last BCG instillation to recurrence was 9 mo (IQR 5–28). The highest stage before HIVEC was pTa in 38 (43.2%), pT1 in 39 (44.3%), and CIS only in 11 (12.5%) cases. High-grade disease was present in 77 patients (87.5%) and low-grade disease in 11 (12.5%) before HIVEC. CIS was present before HIVEC in 51 (58.0%) and at initiation of HIVEC in 47 (53.4%) cases. The median EORTC recurrence and progression scores at HVIEC were 6 (IQR 5–9) and 13 (IQR 9–17), respectively. According to the EAU risk stratification, 17 patients (19.3%) had intermediate-risk, 15 (17.1%) had high-risk, and 56 (63.6%) had very high-risk disease at the time of HIVEC. The median number of HIVEC instillations was six (IQR 6–10). Sixteen patients (18.2%) discontinued treatment during induction, 19 (21.6%) completed induction and maintenance (12 instillations), and 53 (60.2%) discontinued treatment after induction or during maintenance. Reasons for discontinuation were disease recurrence/persistence (16/72, 22.2%) or side effects/unwillingness (56/72, 77.8%; Table 1). Despite the high rate of side effects, no grade >2 adverse events were observed.

**Table 1:** Patient characteristics

Parameter	Result
Patients (*n*)	88
Median follow-up, mo (IQR)	38 (22–53)
Median age, yr (IQR)	71 (66–77)
Sex, *n* (%)	
Male	75 (85.2)
Female	13 (14.8)
Reason for HIVEC, *n* (%)	
BCG failure (second line)	65 (73.9)
Other (first line)	23 (26.1)
BCG failure status, *n* (%)	
No BCG failure	23 (26.1)
BCG-relapsing disease	42 (47.7)
BCG-refractory disease	23 (26.1)
Highest tumor stage before HIVEC, *n* (%)	
pTa	38 (43.2)
pT1	39 (44.3)
pTis only	11 (12.5)
Highest grade (2004/2016) before HIVEC, *n* (%)	
Low grade	11 (12.5)
High grade	77 (87.5)
CIS before HIVEC, *n* (%)	
Yes	51 (58.0)
No	37 (42.0)
Median EORTC recurrence score (IQR)	6 (5–9)
Median EORTC progression score (IQR)	13 (9–17)
EAU NMIBC risk category at HIVEC, *n* (%)	
Intermediate-risk	17 (19.3)
High-risk	15 (17.1)
Very high-risk	56 (63.6)
Median number of HIVEC instillations, *n* (IQR)	6 (6–10)
HIVEC discontinuation status, *n* (%)	
No discontinuation	19 (21.6)
Discontinuation during induction cycle	16 (18.2)
Discontinuation during maintenance cycle	53 (60.2)

BCG = bacillus Calmette-Guérin; IQR = interquartile range; HIVEC = hyperthermic intravesival chemotherapy; CIS = carcinoma in situ; EORTC = European Organization for Research and Treatment of Cancer; EAU = European Association of Urology; NMIBC = non–muscle-invasive bladder cancer.

### 3.2 Primary endpoint

**Fig. 1:**
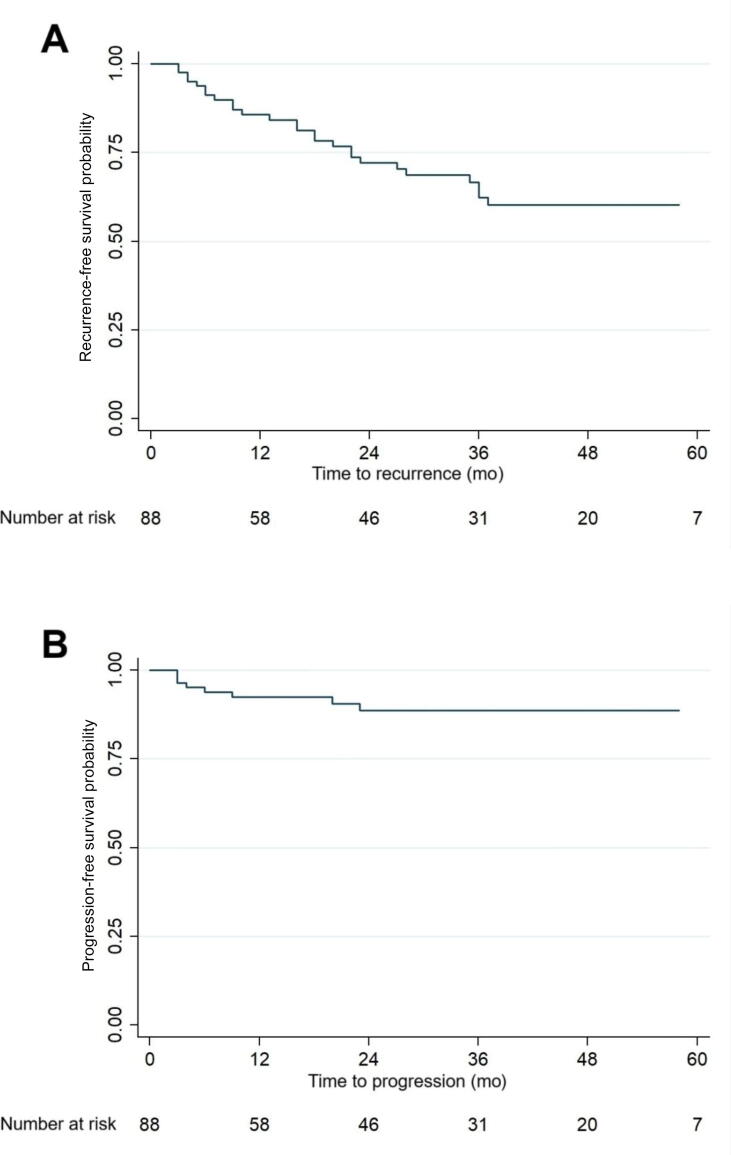
Kaplan-Meier curves for overall (A) intravesical recurrence-free survival and (B) progression-free-survival.

Twenty-six patients (29.6%) experienced intravesical recurrence (Fig. 1A). Of 26 patients, recurrence stages were recurrent/persistent CIS in nine (34.6%), low-grade pTa in three (11.5%), high-grade pTa in five (19.2%), high-grade pT1 in five (19.2%), and ≥pT2 in four (15.4%) cases. While 24/65 patients (36.9%) treated with HIVEC after BCG failure experienced intravesical recurrence, only 2/23 (8.7%) treated with HIVEC in a first-line setting developed intravesical recurrence.

**Fig. 2:**
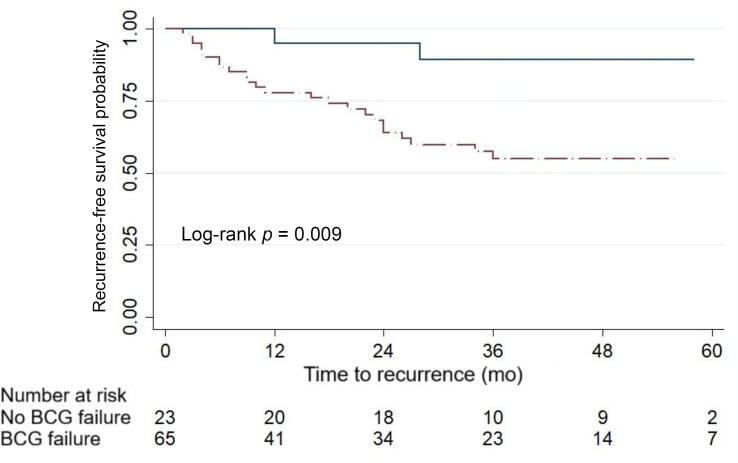
Kaplan-Meier curve for survival free from intravesical recurrence for patients with (red) and without (blue) bacillus Calmette-Guérin (BCG) failure. (For interpretation of the references to colour in this figure legend, the reader is referred to the web version of this article).

**Fig. 3:**
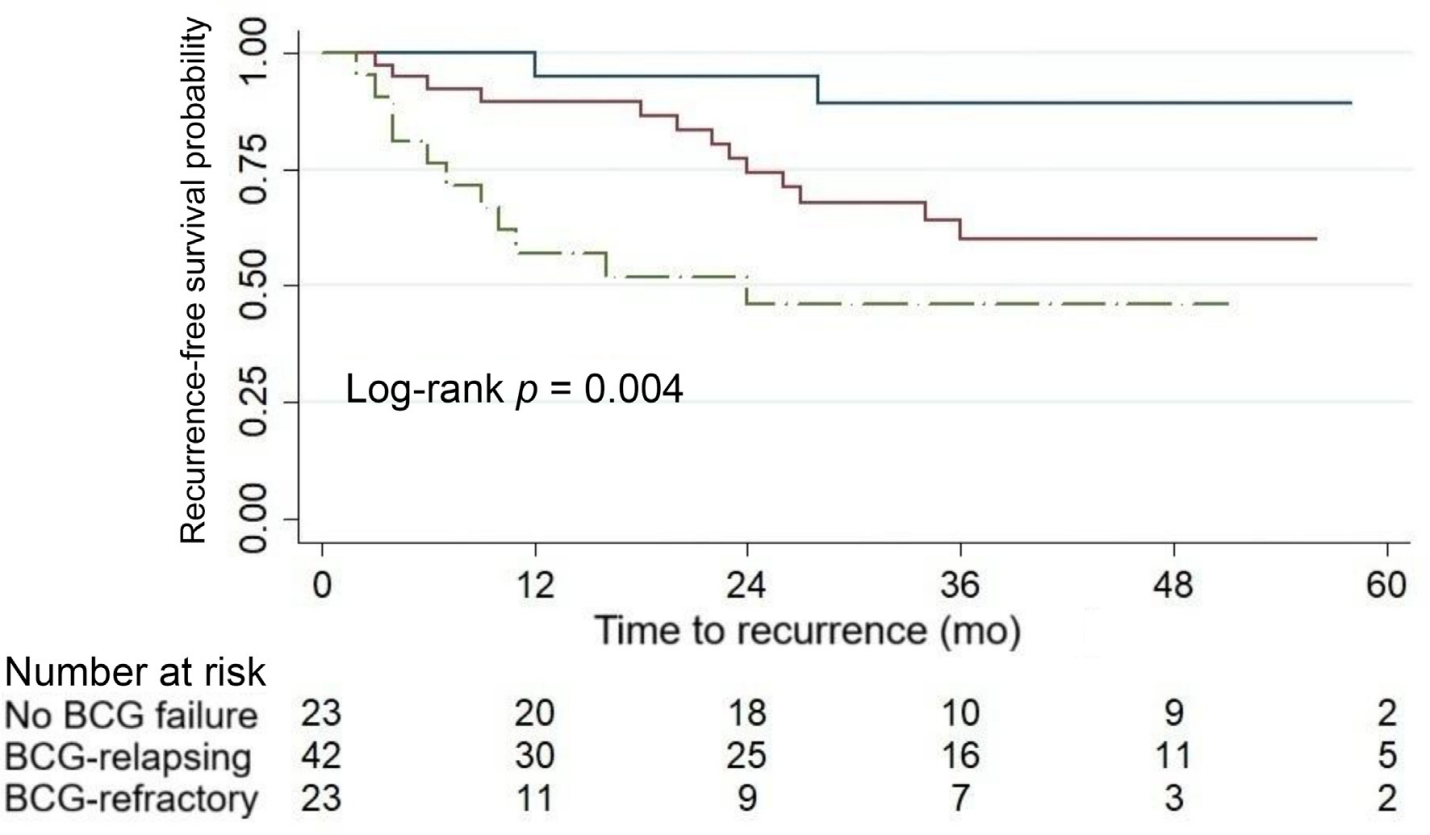
Kaplan-Meier curve for survival free from intravesical recurrence for patients without BCG failure (blue) and those with BCG-relapsing (red) and BCG-refractory (green) tumors. BCG = bacillus Calmette-Guérin. (For interpretation of the references to colour in this figure legend, the reader is referred to the web version of this article).

In the overall cohort, median intravesical RFS was 29 mo (IQR 10–48) with 1-yr and 2-yr intravesical RFS rates of 84.1% and 77.3%, respectively. Median intravesical RFS for the group of patients without recurrence was 39 mo (IQR 21–53). Kaplan-Meier curves and log-rank tests showed significantly lower RFS in the group with CIS before HIVEC (*p* = 0.006), the group with BCG failure (*p* = 0.009; Fig. 2), and the group with BCG-refractory tumors (*p* = 0.004; Fig. 3).

Univariate Cox regression analysis revealed that CIS before HIVEC (*p* = 0.011), higher EAU NMIBC risk category (*p* = 0.036), BCG failure (HR 5.50, 95% CI 1.30–23.30; *p* = 0.021), and BCG-refractory tumors (HR 2.55, 95% CI 1.43–4.55; *p* = 0.001) were associated with worse intravesical RFS (Table 2). Multivariable Cox regression analysis also revealed that BCG-refractory tumors were associated with worse RFS (HR 2.22, 95% CI 1.15–4.29; *p* = 0.018; Table 3). The proportional-hazards assumption was not violated, as the global test for the multivariable Cox model was nonsignificant (*p* = 0.539).

**Table 2:** Univariate Cox regression results for survival free from intravesical recurrence

Variable	HR (CI 95%)	*p* value
Age	0.91 (0.42–1.98)	0.810
Sex	1.46 (0.55–3.88)	0.446
Number of recurrences before HIVEC	1.21 (0.96–1.47)	0.056
CIS before HIVEC	3.75 (1.35–9.49)	**0.011**
Highest tumor stage before HIVEC	1.54 (0.88–2.71)	0.133
Highest tumor grade before HIVEC	3.81 (0.65–22.3)	0.139
EORTC recurrence score at HIVEC	1.20 (0.62–2.34)	0.590
EAU risk category at HIVEC	2.20 (1.05–4.61)	**0.036**
BCG failure	5.50 (1.30–23.30)	**0.021**
BCG-refractory tumor	2.55 (1.43–4.55)	**0.001**

HR = hazard ratio; CI = confidence interval; HIVEC = hyperthermic intravesical chemotherapy; CIS = carcinoma in situ; EORTC = European Organisation for Research and Treatment of Cancer; EAU = European Association of Urology; BCG = bacillus Calmette-Guérin.

**Table 3:** Multivariable Cox regression results for survival free from intravesical recurrence

Variable	HR (CI 95%)	*p* value
Age	0.73 (0.33–1.64)	0.447
Sex	1.31 (0.49–3.54)	0.590
CIS before HIVEC	1.56 (0.59–4.08)	0.369
BCG-refractory tumor	2.22 (1.15–4.29)	**0.018**

CIS = carcinoma in situ; HIVEC = hyperthermic intravesical chemotherapy; BCG = bacillus Calmette-Guérin.

### 3.3 Secondary endpoints

Eight patients (9.1%) experienced progression, four of whom (50.0%) had metastatic disease at the time of intravesical recurrence, and seven (87.5%) had metastatic disease by the end of follow-up (Fig. 1B). Median PFS was 38 mo (IQR 21–53), with 1-yr and 2-yr PFS rates of 96.6% and 93.2%, respectively. Median PFS for the group of patients without progression was 39 mo (IQR 24–55). Kaplan-Meier curves and log-rank tests revealed significantly shorter PFS for the group with BCG-refractory tumors before HIVEC (*p* = 0.011), although univariate and multivariable Cox regression analysis did not identify any factors significantly associated with progression.

Twenty-one patients (23.9%) experienced extravesical recurrence, defined as urothelial cancer in the upper urinary tract (*n* = 11, 52.4%), prostatic urethra (*n* = 5, 23.8%), or both (*n* = 5, 23.8%). Of these, six patients (28.6%) developed locoregional and/or distant metastatic disease during further follow-up. Median extravesical RFS was 29 mo (IQR 10–49) overall, and 35 mo (IQR 19–53) for censored patients. Kaplan-Meier curves and log-rank tests revealed significantly shorter extravesical RFS for the groups with CIS before HIVEC (*p* = 0.014), high-grade tumors at the time of HIVEC (*p* = 0.019), higher EAU risk category at the time of HIVEC (*p* = 0.007), BCG failure (*p* = 0.002), and BCG-refractory tumors before HIVEC (*p* = 0.003). Univariate Cox regression analysis revealed that CIS before HIVEC (*p* = 0.022) was associated with shorter extravesical RFS.

### 3.4 Exploratory endpoints

A total of 34 patients (38.6%) experienced recurrence in the urinary tract, with median DFS of 27 mo (IQR 8–44) and 1-yr and 2-yr DFS rates of 65.9% and 52.3%, respectively. Seven deaths were cancer-related, resulting in median CSS of 42 mo (IQR 25–48). A total of 24 patients (27.3%) died during follow-up, resulting in median OS of 38 mo (IQR 22–54). Univariate Cox regression analysis revealed that age was the only factor associated with shorter OS (*p* = 0.035).

Thirteen patients (14.8%) had to undergo cystectomy because of either recurrent disease (*n* = 8, 9.1%), progression to muscle-invasive disease (*n* = 3, 3.4%), or functional reasons (*n* = 2, 2.3%), resulting in a bladder preservation rate of 85.2%.

## 4 Discussion

In our two-center study investigating epirubicin-based HIVEC in 88 patients with NMIBC, the majority categorized as having EAU very high-risk (*n* = 56, 63.6%), we observed 26 intravesical recurrences (29.6%), with median RFS of 29 mo (IQR 10–48) and 1-yr and 2-yr RFS rates of 84.1% and 77.3%, respectively. When considering just the BCG-unresponsive group (1-yr and 2-yr RFS rates of 61.3% and 54.8%), our results substantially exceed thresholds defined by the International Bladder Cancer Group for emerging therapies considered clinically meaningful, at RFS rates of 50% at 6 mo, 30% at 12 mo, and 25% at 18 mo for papillary tumors [32]. We also observed median PFS of 38 mo (IQR 21–53), with 1-yr and 2-yr PFS rates of 96.6% and 93.2%, respectively. Importantly, even after expanding our initial BCG failure cohort by 49 patients and extending median follow-up from 28 to 38 mo, promising long-term results were maintained [31]. Among all the significant factors, BCG-refractory disease showed the strongest association with intravesical recurrence (univariate: HR 2.55, 95% CI 1.43–4.55; *p* = 0.001; multivariable: HR 2.22, 95% CI 1.15–4.29; *p* = 0.018), while the associations with intravesical recurrence for CIS before HIVEC (*p* = 0.011) and BCG failure (*p* = 0.021) should be considered more exploratory.

In comparison to recent studies using MMC-based HIVEC, our outcomes appear to be promising. Kastner et al [33] reported 1-yr and 2-yr RFS rates of 67% and 40%, and a 2-yr PFS rate of 98.0%, while Pignot et al [34] reported 1-yr and 2-yr RFS rates of 62.9% and 36.8%, and 1-yr and 2-yr PFS rates of 92.2% and 87.9%, respectively. Although our study design does not allow for a direct comparison, the higher RFS rates in our study may be explained by the use of epirubicin instead of MMC, particularly as HIVEC with epirubicin has rarely been studied [35], [36], [37]. However, the limited evidence available from comparisons of epirubicin and MMC, based on small patient numbers, has not demonstrated significant oncological differences [35], [36]. Another explanation could be our optimized treatment protocol, which included fluid restriction to minimize drug dilution and urine alkalinization to minimize biodegradation.

We also observed a relatively high number of extravesical recurrences (*n* = 21, 23.9%), involving the upper urinary tract (11/21, 52.4%), prostatic urethra (five of 21, 23.8%), or both (five of 21, 23.8%). In nine patients (10.2%), extravesical recurrence occurred in conjunction with intravesical recurrence. Direct comparison with other studies is limited, as extravesical recurrences were not reported separately [33], [34], [35], [36], [37]. In our study, CIS before HIVEC was significantly related to extravesical recurrence on univariate analysis. Furthermore, CIS before HIVEC, high-grade tumor at HIVEC, higher EAU risk category at HIVEC, BCG failure, and BCG-refractory disease were significantly associated with shorter extravesical RFS. This highlights again that urothelial carcinoma is a panurothelial disease that must be considered in follow-up [38]. BCG topical agents need contact with the tumor to be effective. In our cohort, extravesical recurrences were only observed in patients treated after prior BCG exposure or failure (none in BCG-naïve patients), which suggests an association with baseline tumor history. However, given the single-arm design, attribution to HIVEC cannot be determined. Precise information on tumor locations and surgical procedures would be of interest in this regard; however, the data available were insufficient for inclusion in this analysis.

Sequential instillation of gemcitabine and docetaxel, which was first described in 2015 during BCG and MMC shortages [39], is another emerging treatment for patients with BCG failure. This regimen has recently been included in guidelines, although with a weak recommendation because of limited prospective evidence [4]. Steinberg et al [40] reported 1-yr and 2-yr RFS rates of 60.0% and 46.0%, respectively, with tumor progression in ten patients (3.6%) in a cohort of 276 patients with BCG-pretreated NMIBC. McElree et al [41] investigated gemcitabine and docetaxel for first-line treatment because of BCG shortages in a cohort of 107 BCG-naïve patients with high-risk disease. The 1-yr and 2-yr RFS rates were 85.0% and 82.0%, respectively, with no progression or cancer-related deaths. Taken together, these results suggest comparable oncological outcomes, and possibly even more favorable RFS, for epirubicin-based HIVEC in an optimized treatment setting.

Our study has several limitations. First, the cohort was relatively small and heterogeneous in terms of tumor history, including stage, grade, number of recurrences, and the interval between BCG and HIVEC, with a possibility of confounding. The inclusion of patients who received HIVEC as first-line therapy introduced a subgroup with demonstrably superior oncological outcomes that warrants separate analysis in future studies. Second, 16 patients (18.2%) discontinued HIVEC during the induction cycle, and 53 (60.2%) did not start or complete maintenance therapy, which complicates intracohort comparisons and is a possible source of attrition bias that could potentially affect survival estimates. The high discontinuation rate further raises uncertainty regarding potential oncological outcomes with consistent adherence to therapy, as the majority of treatment discontinuations (77.8%) were related to side effects and unwillingness to continue treatment. Nevertheless, unlike BCG maintenance therapy, evidence supporting maintenance instillation with chemotherapeutic agents remains inconclusive [42].

Our multivariable models were constructed using univariate screening (*p* ≤ 0.05) to select candidate variables, in addition to variables selected a priori. A limitation of this approach is a higher risk of overfitting, which can lead to unstable coefficient estimates and confidence intervals. The multivariable analysis results should therefore be interpreted with caution, and all associations identified should be considered as exploratory. In view of the observational design, the multivariable associations may still be influenced by residual confounding (eg, inclusion of lower-risk cases) and other sources of bias. Given the observational design, the heterogeneous cohort, and the absence of a controlled comparator, the analysis is also associative rather than causal. Potential sources of bias include selection bias due to treatment allocation on the basis of clinical judgment, and information bias arising from retrospective data analysis. These sources of bias were mitigated by the use of prospectively maintained databases and predefined inclusion criteria. Furthermore, the generalizability of our study is limited by the single-arm design and the heterogeneity of tumor characteristics. Another key limitation is incomplete accounting for participants. This retrospective analysis used a prospectively maintained database that captured consecutive HIVEC-treated patients but did not reliably record the number assessed for eligibility or reasons for exclusion; therefore, a complete flow chart of those screened, eligible, and excluded cannot be provided.

Nevertheless, this update strengthens our initial report by expanding patient numbers, extending follow-up, and confirming encouraging oncological outcomes. To the best of our knowledge, this represents the largest study of patients treated with epirubicin-based HIVEC to date. Strengths include the well-defined treatment protocol, prospective data collection, and multicenter design, all of which underline the clinical relevance of our study.

## 5 Conclusions

In this multicenter retrospective cohort, epirubicin-based HIVEC was associated with encouraging oncological outcomes in both BCG-naïve patients and those with BCG failure. These findings indicate that epirubicin-based HIVEC could be a potential treatment alternative for patients with BCG failure who are unfit for, or decline, radical cystectomy, and support its consideration as first-line therapy in the context of BCG shortages or contraindications. Extravesical recurrences occurred at a relatively high rate in the BCG failure group, which underscores the need for monitoring for extravesical disease in future studies and follow-up strategies. Because of the single-arm design, heterogeneity of the cohort, and potential confounding, our findings should be interpreted with caution and viewed as hypothesis-generating. Comparative prospective studies are needed to determine the relative effectiveness of epirubicin-based HIVEC.

  ***Author contributions***: Nicolas Arnold had full access to all the data in the study and takes responsibility for the integrity of the data and the accuracy of the data analysis.

  *Study concept and design*: Roth.

*Acquisition of data*: Arnold, Koneval, Blanc.

*Analysis and interpretation of data*: Vartolomei, Arnold, Koneval.

*Drafting of the manuscript*: Arnold, Koneval.

*Critical revision of the manuscript for important intellectual content*: Kiss, Schneidewind, Giudici, Thalmann, Lucca.

*Statistical analysis*: Vartolomei.

*Obtaining funding*: None.

*Administrative, technical, or material support*: None.

*Supervision*: Roth.

*Other*: None.

  ***Financial disclosures:*** Nicolas Arnold certifies that all conflicts of interest, including specific financial interests and relationships and affiliations relevant to the subject matter or materials discussed in the manuscript (eg, employment/affiliation, grants or funding, consultancies, honoraria, stock ownership or options, expert testimony, royalties, or patents filed, received, or pending), are the following: None.

  ***Funding/Support and role of the sponsor*:** None.
